# Roflumilast for Oral Ulcers in Behçet's Disease and Recurrent Aphthous Stomatitis

**DOI:** 10.1111/1346-8138.17972

**Published:** 2025-09-23

**Authors:** Kyung Bae Chung, Hae June Sung, Eun Hye Kim, Hyunwoo Jang, Do‐Young Kim

**Affiliations:** ^1^ Department of Dermatology, Cutaneous Biology Research Institute Yonsei University College of Medicine Seoul Republic of Korea

**Keywords:** Behçet's disease, oral ulcers, PDE4 inhibitor, recurrent aphthous stomatitis, roflumilast

## Abstract

Oral aphthous ulcer in Behçet's disease (BD) and recurrent aphthous stomatitis (RAS) is a cause of discomfort for many patients, especially in cases refractory to colchicine or azathioprine. Roflumilast, a phosphodiesterase‐4 (PDE4) inhibitor, may be effective for treating refractory oral ulcers (OUs) in BD and RAS, especially in regions where apremilast is unavailable. In this study, we investigated the efficacy and safety profile of low‐dose roflumilast for refractory OUs in BD and RAS. This single‐center, single‐arm, retrospective observational study included 46 patients screened from the outpatient department from May 2023 to Dec 2023. During the 12‐week study period, the subjects received roflumilast at a dosage of 0.25 mg daily. For those experiencing adverse events (AEs) requiring adjustment, the dose was reduced to 0.125 mg. Objective clinical responses were evaluated as clinician‐assessed treatment categories (complete remission, partial response, or non‐response), based on the absence or presence of new OUs and symptom improvement. Subjective symptoms were evaluated through a patient‐reported questionnaire, and AEs were monitored through the protocol. At week 12, 71.7% of patients showed a positive response to roflumilast, with 30.4% achieving complete remission. AEs were reported in 76.1% of the 46 subjects with follow‐up visits, primarily gastrointestinal (71.7%) and neurological symptoms (17.4%). Among the cohort, 78.3% of patients tolerated roflumilast without discontinuation, including 15.2% with dose reduction, while 21.7% discontinued due to intolerable AEs. Roflumilast demonstrated rapid and sustained efficacy in reducing OUs in BD and RAS. Although AEs were frequent, they were generally tolerable and manageable. While the study has limitations, including its retrospective observational nature and small sample size, it suggests roflumilast as a potential treatment alternative for refractory OUs where apremilast is unavailable, deserving further research.

## 1 Introduction

Oral aphthae in patients with Behçet's disease (BD) and recurrent aphthous stomatitis (RAS) cause significant discomfort and require long‐term management [1, 2]. While colchicine or azathioprine is a common choice for systemic treatment of refractory oral ulcers, some patients show insufficient response or intolerance [3]. Apremilast, a phosphodiesterase‐4 (PDE4) inhibitor, has proven to be effective for the treatment of oral ulcers in BD through phase‐3 randomized controlled studies [4, 5, 6], but was unavailable in some high‐prevalence countries, including Turkey and Korea, at the time of the study [7]. Roflumilast, another PDE4 inhibitor approved for chronic obstructive pulmonary disease (COPD), has similar molecular properties with higher binding affinity to PDE4 and longer plasma halftime [8]. Thus, we investigated the effectiveness and safety of roflumilast as an alternative treatment option for refractory oral ulcers.

## 2 Methods

### 2.1 Study Population

This study is a single‐center, single‐arm, retrospective, observational study conducted over a period of 12 weeks. This study was approved by the Institutional Review Board (IRB, number: 4‐2023‐1427) at Yonsei University and was conducted in accordance with the principles expressed in the Declaration of Helsinki. We retrospectively reviewed 46 patients treated with roflumilast for refractory oral ulcers. In this study, refractory oral ulcers were defined as those occurring in patients with BD or RAS, who experienced ≥ 3 episodes of oral ulcers in the preceding 12 months and did not respond adequately to, or were intolerant of, at least 3 months of colchicine‐based conventional treatment (0.6–1.2 mg daily), with or without additional immunosuppressive agents such as azathioprine (≥ 50 mg/day), or were intolerant of such treatment. These patients experienced at least two new oral ulcers during the previous month despite the use of colchicine ranging from 0.6 to 1.2 mg per day, possibly in combination with other immunosuppressive agents such as azathioprine (over 50 mg/day). Patients with active major organ involvement related to BD that required systemic rescue treatment were excluded from the study. Patients with prior biologic use or significant medical conditions were excluded. Despite the study's retrospective nature, it was rigorously executed according to a predefined standard care flow. In our structured clinical care environment, all patients attended scheduled visits throughout the 12‐week period, with no unplanned loss to follow‐up. However, 10 patients (21.7%) discontinued roflumilast treatment due to intolerable adverse events while continuing clinic visits for safety monitoring.

### 2.2 Outcome Measures

Clinical effectiveness and safety were assessed at baseline, weeks 4, 8, and 12. The primary outcome was categorized into complete remission (CR) for no new oral ulcers in the last 4 weeks, partial response (PR) for improved symptoms but with new oral ulcers, and non‐responder (NR) for no improvement. Secondary outcomes included ulcer activity, assessed through a patient questionnaire on lesion count, total new lesions in the last 4 weeks, lesion duration of a single lesion, persistent duration of overall oral ulcer, and pain severity using a numeric rating scale (NRS). In addition, modified composite index scores for oral ulcer activity were retrieved [9]. Safety was measured by tracking adverse events (AEs) after initiation, with discontinuation recorded as intolerable AEs. Outcome measures were written in a standardized, predetermined medical record format.

## 3 Results

### 3.1 Baseline Characteristics

Our cohort included 46 patients with a mean age of 52.4 (SD, 13.0) (Table 1). Thirty‐seven (80.4%) patients were diagnosed as BD, and 9 (19.6%) had RAS without matching diagnostic criteria for BD. Participants received treatment with roflumilast at a starting dose of 0.25 mg (half tablet) per day, with adjustment depending on adverse events and patient tolerability. Initially, when roflumilast was administered, there were no modifications to dosages of existing medications, including colchicine or azathioprine; however, dose reductions were permitted in cases of symptom improvement.

**TABLE 1:** Baseline demographics of study population.

Baseline characteristics, no. (%)	Total subjects (*n* = 46)
Gender	
Male	19 (41.3%)
Age (mean, SD)	52.4 (13.0)
Diagnosis	
BD	37 (80.4%)
RAS	9 (19.6%)
HLA‐B51 typing	
Negative	17 (37.0%)
Positive	8 (17.4%)
Not available	21 (45.7%)
Colchicine treatment history	
Insufficient efficacy	43 (93.5%)
Intolerance	1 (2.2%)
Contraindicated	2 (4.4%)
Concurrent medication usage	
Colchicine	34 (73.9%)
Azathioprine	2 (4.4%)
Colchicine and azathioprine	7 (15.2%)
Colchicine and cyclosporine	1 (2.2%)
Colchicine and cyclosporine and azathioprine	1 (2.2%)
Others	1 (2.2%)
Oral ulcer lesion activity (mean, SD)	
The number of current lesions	1.4 (1.2)
The number of total lesions appeared during last 4 weeks	4.4 (3.4)
Average duration of lesions (days)	9.8 (5.1)
Lesion‐free days during last 4 weeks (days)	19.7 (7.3)
Pain (NRS)	5.2 (1.7)

Abbreviations: BD, Behçet's disease; NRS, Numeric rating scale; RAS, recurrent aphthous stomatitis.

### 3.2 Primary Outcome

**FIGURE 1:**
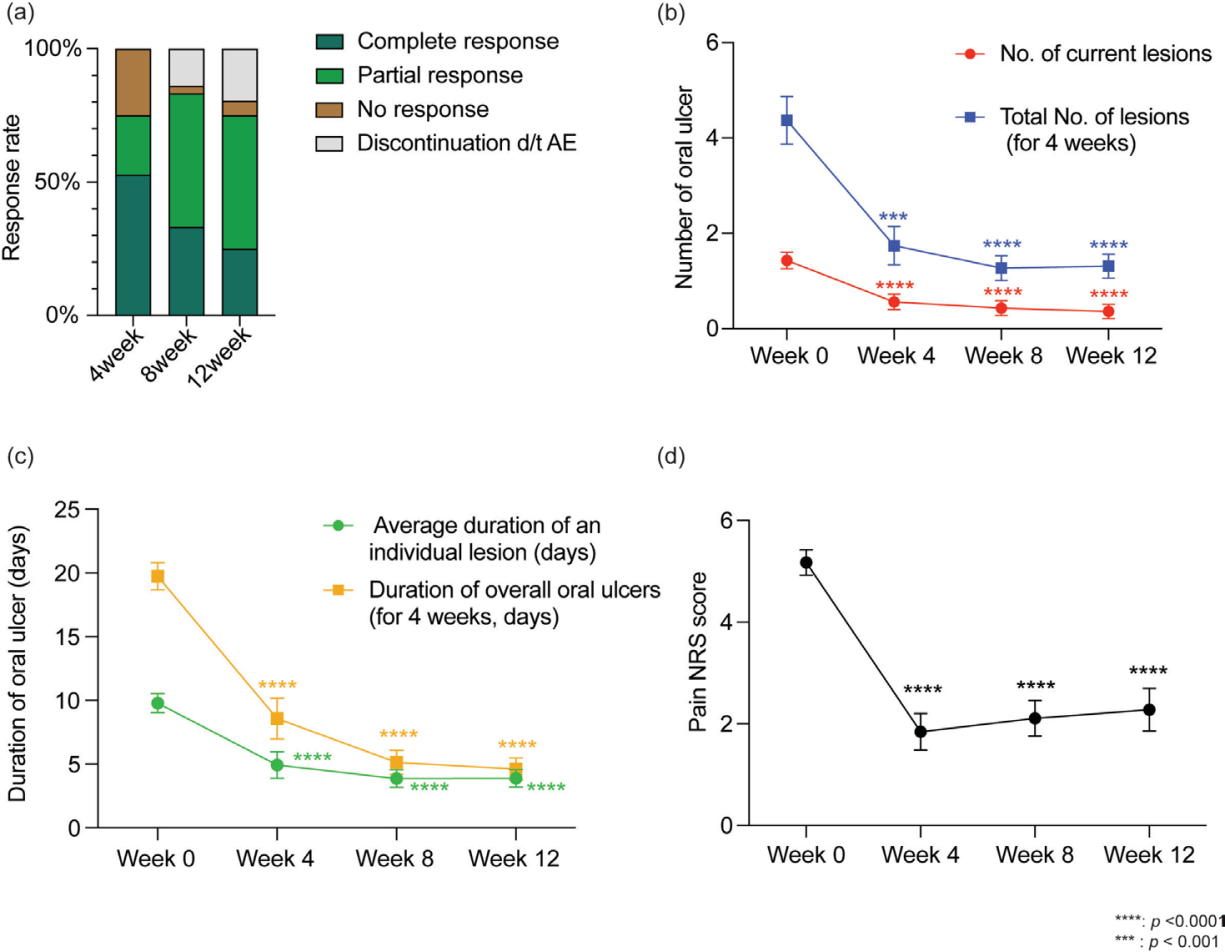
Clinical efficacy of roflumilast in BD and RAS. The roflumilast treated population included all the patients who received at least one dose of roflumilast. The error bars indicate the standard error. (a) Response rate evaluated at weeks 4, 8 and 12. (b) Number of oral ulcers. (c) Duration of oral ulcer. (d) Pain score evaluated by NRS score at each evaluation. Statistical comparison was evaluated compared to baseline (week 0) using multiple comparison.

At 4 weeks, 34 patients (74.0%) responded to roflumilast, including 21 (45.7%) with CR and 13 (28.3%) with PR. Nine patients (19.6%) showed no response (Figure 1a, Table S1). By week 8, 35 out of 46 (76.1%) responded, with 14 (30.4%) achieving CR and 21 (45.7%) PR. Only 2 (4.4%) patients showed no response, and 9 (19.6%) discontinued due to intolerable AEs (Table S1). At 12 weeks, both intention‐to‐treat (ITT) and per‐protocol (PP) analyses were performed. In the ITT analysis, 33 of the 46 (71.7%) remained as responders, comprising 14 (30.4%) with CR and 19 (41.3%) with PR. In the PP analysis of the 36 patients who completed 12 weeks of treatment, the response rate was 91.7% (33/36), with 14 (38.9%) achieving CR and 19 (52.8%) achieving PR. Additionally, 3 patients (6.5%) were NR, and 10 (21.7%) discontinued due to intolerance.

### 3.3 Secondary Outcome

At baseline, the mean (SD) number of existing lesions of oral ulcer was 1.44 (SD, 1.21). The total number of lesions that occurred during the previous 4 weeks from baseline was 4.37 (SD, 3.40) (Figure 1b, Table S1). The average duration of each lesion was 9.78 (SD, 5.10) days, and the overall oral ulcer persistent period during the last 4 weeks was 19.74 (SD, 7.26) days (Figure 1c). The average NRS pain score was 5.17 (SD, 1.70) (Figure 1d). Compared to the baseline visit, a decreasing trend of disease activity at weeks 4 and 8 throughout all scales, including the number, duration, and pain of lesions, was identified. At week 12, indices for disease activities showed a slight increment compared to week 8, yet remained relatively low compared to the baseline. The composite index score, which is based on both oral ulcer activity and pain, also shows statistically significant improvement from week 4 through 12 (Figure S1) [9]. Subgroup analysis revealed no significant differences in the improvement of oral ulcers between the RAS and BD groups (Figure S2).

### 3.4 Adverse Events

A total of 35 (76.1%) subjects complained of adverse events (Table 2). The most common adverse event was gastrointestinal (GI) symptoms, with 33 (71.7%) subjects including loose stool, diarrhea, or urge to defecate. Eight patients (17.4%) suffered from neurological symptoms, including insomnia (4.3%) and numbness of extremities (4.3%). While twenty‐eight (60.9%) subjects maintained the initial dose of 0.25 mg, 7 (15.2%) subjects needed a dose reduction to continue the treatment. Meanwhile, 10 (21.7%) subjects failed to continue the treatment due to intolerable adverse events, including gastrointestinal symptoms (9 subjects, 19.6%) and neurological symptoms (2 subjects, 4.3%). However, all recovered after stopping roflumilast for 4 weeks, with no severe adverse events reported.

**TABLE 2:** Adverse events after administration of roflumilast.

Adverse events, no. (%)	Patients (*n* = 46)
Total subjects with adverse events	35 (76.1%)
Tolerable without discontinuation	36 (78.3%)
Tolerable without dose adjustment	28 (60.9%)
Tolerable after dose adjustment	7 (15.2%)
Gastrointestinal symptoms	33 (71.7%)
Loose stool/diarrhea/feeling urge to defecate	29 (63.0%)
Abdominal discomfort/abdominal pain	8 (17.4%)
Nausea	3 (6.5%)
Neurological symptoms	8 (17.4%)
Insomnia	2 (4.3%)
Numbness of extremities	2 (4.3%)
Headache	2 (4.3%)
Dizziness	1 (2.2%)
Tinnitus	1 (2.2%)
Tremor	1 (2.2%)
General symptoms	2 (4.3%)
Loss of appetite/weight loss	2 (4.3%)
Intolerance requiring discontinuation	10 (21.7%)
Gastrointestinal symptoms	9 (19.6%)
Neurological symptoms	2 (4.3%)
Both gastrointestinal and neurological symptoms	1 (2.2%)

## 4 Discussion

This study suggests a notable decrease in oral ulcers with the administration of low‐dose roflumilast in BD and RAS. The efficacy of roflumilast (71.7%–76.1%) is comparable to that of 76% in a phase 3 study of apremilast for BD [4]. Though the mean number of oral ulcers in our cohort was lower than previously reported (1.43 vs. 4.2 at baseline), likely reflecting milder disease activity in our real‐world setting compared to controlled trials during active flares, the proportional reduction mirrors that seen with apremilast. This supports roflumilast as a viable treatment for oral ulcers where apremilast is unavailable. Our cohort demonstrated pronounced effects early on, which gradually attenuated. This pattern may reflect natural disease fluctuations and our strict definition of complete remission, as well as a potential influence from tapering of concurrent immunomodulators. Thus, future research should control the use of other medications more strictly. Additionally, since a direct comparison of the effects of this medication with apremilast is not substantiated, further studies, including randomized controlled trials, are required. Adverse events occurred in 76.1% of participants, mainly gastrointestinal and neurological symptoms, yet a majority (78.3%) continued treatment for 12 weeks, indicating a generally tolerable side effect profile. The discontinuation rate of 21.7% was lower than the 28%–30% reported in the real‐world use of apremilast for BD and other skin disorders [10, 11]. This may be due to a shorter follow‐up period and initiation with a low dosage. All adverse events resolved after stopping roflumilast, without severe adverse events. Patients with adverse events did not show an increase in any laboratory inflammatory markers and were not diagnosed as GI BD nor neuro‐BD. This suggests that the symptoms were likely attributable to the roflumilast. Interestingly, while our cohort had a higher incidence of diarrhea (63.0%), neurological side effects such as headaches were less frequent compared to previous studies [4]. However, the occurrence of numbness of extremities, which was not reported previously, might be associated with the concurrent administration of other immunosuppressants like colchicine, known to cause gastrointestinal issues and neuropathy [12]. The incidence of AEs in COPD patients was lower, suggesting single regimen administration might reduce side effects [8]. Hwang et al. reported that a dose‐escalation regimen of roflumilast from 0.25 to 0.5 mg for COPD patients can minimize the risk of intolerable adverse events [13]. Therefore, in this study, we administered low‐dose roflumilast (0.25 mg/day) to patients to mitigate AEs. Notably, two recent single‐arm observational studies from Spain also demonstrated efficacy in BD and RAS, but involved smaller cohorts [14, 15]. In contrast, our study included a larger patient cohort and was conducted in a real‐world setting where roflumilast was added to ongoing colchicine treatment, better reflecting routine clinical practice for refractory oral ulcers. Although its retrospective design, lack of a control group, and small, ethnically homogeneous cohort are limitations, the findings support roflumilast as a potential treatment option in settings where apremilast is inaccessible.

## Conflicts of Interest

The authors declare no conflicts of interest.

## Supporting information


**Figure S1:** Clinical efficacy of roflumilast evaluated with modified oral ulcer composite index. Change in modified oral ulcer composite index calculated by the sum of oral ulcer activity (0–1 points) and pain status (0–5 points) based on Mumcu et al.'s scale.
**Figure S2:** Clinical efficacy of roflumilast comparing BD and RAS group. The roflumilast treatment outcomes comparing BD and RAS group. The error bars indicate the standard error. (a) Response rate evaluated at weeks 4, 8 and 12. (b) Number of current lesions at the visit. (c) Total number of oral ulcers during 4‐week period. (d) Average duration of an individual oral ulcer lesion. (e) Total duration of overall oral ulcers within 4‐week period. (f) Pain score evaluated by NRS score at each evaluation. Statistical comparison was evaluated comparing BD and RAS group for each week 4, 8 and 12. (Abbreviations: BD, Behçet's disease; NRS, numeric rating scale; NS, no statistical difference; RAS, recurrent aphthous stomatitis).
**Table S1:** Treatment outcomes after administration of roflumilast.

## Data Availability

The data that support the findings of this study are available from the corresponding author upon reasonable request.
