# Supplementary material for: Roflumilast for Oral Ulcers in Behçet's Disease and Recurrent Aphthous Stomatitis
Source: J Dermatol. 2025 Sep 23;52(11):1713–7. doi: 10.1111/1346-8138.17972 (PMC12592591; doi:10.1111/1346-8138.17972)
Supplement: Supplementary file 1 — Figure S1: Clinical efficacy of roflumilast evaluated with modified oral ulcer composite index. Change in modified oral ulcer composite index calculated by the sum of oral ulcer activity (0–1 points) and pain status (0–5 points) based on Mumcu et al.'s scale. Figure S2: Clinical efficacy of roflumilast comparing BD and RAS group. The roflumilast treatment outcomes comparing BD and RAS group. The error bars indicate the standard error. (a) Response rate evaluated at weeks 4, 8 and 12. (b) Number of current lesions at the visit. (c) Total number of oral ulcers during 4‐week period. (d) Average duration of an individual oral ulcer lesion. (e) Total duration of overall oral ulcers within 4‐week period. (f) Pain score evaluated by NRS score at each evaluation. Statistical comparison was evaluated comparing BD and RAS group for each week 4, 8 and 12. (Abbreviations: BD, Behçet's disease; NRS, numeric rating scale; NS, no statistical difference; RAS, recurrent aphthous stomatitis). Table S1: Treatment outcomes after administration of roflumilast. [file JDE-52-1713-s001.docx]

**Supplementary information**

**Supplemental Figure 1. Clinical efficacy of roflumilast evaluated with modified oral ulcer composite index**

Change in modified oral ulcer composite index calculated by the sum of oral ulcer activity (0-1 points) and Pain status (0-5 points) based on *Mumcu et al*’s scale.


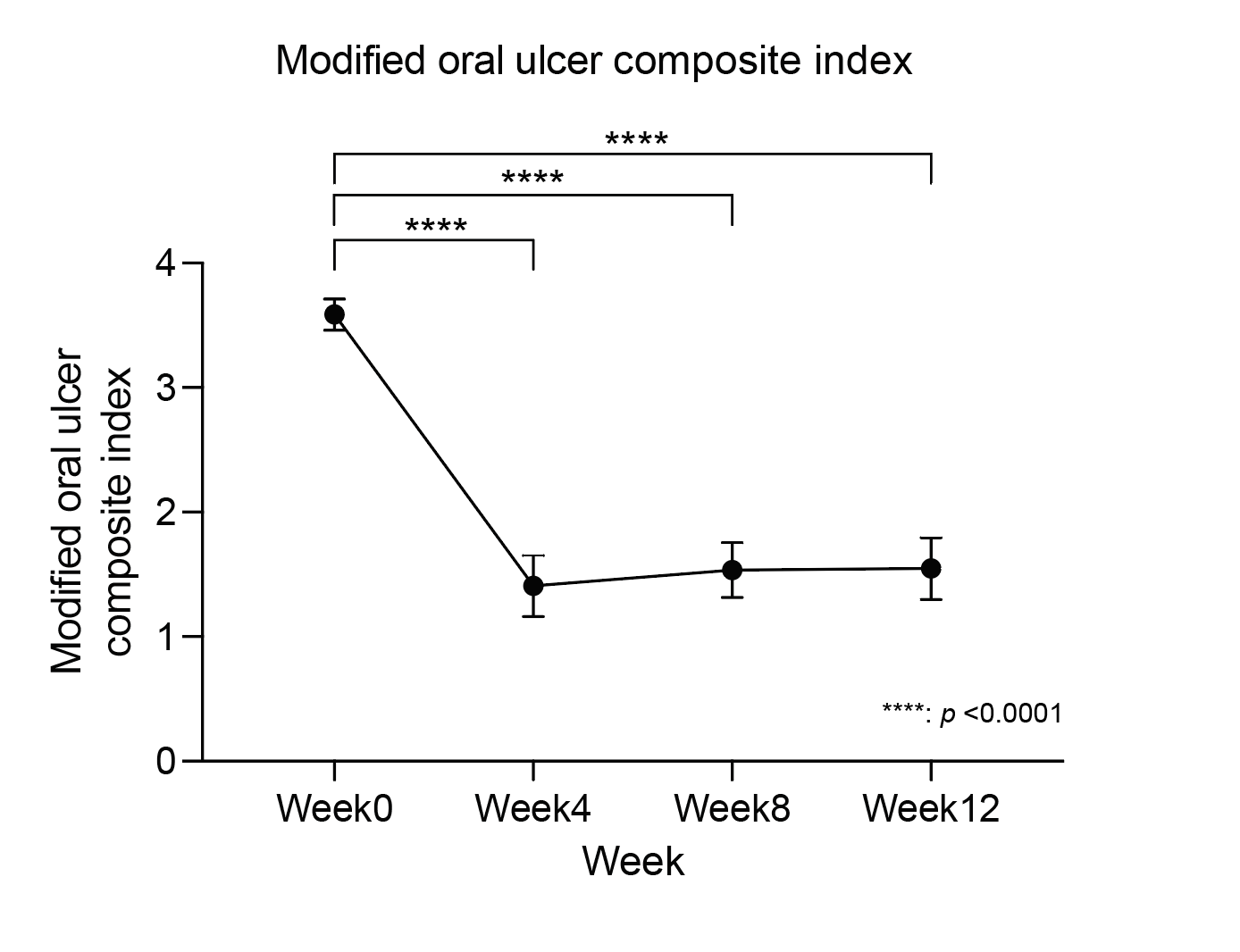


**Supplemental Figure 2. Clinical efficacy of roflumilast comparing BD and RAS group.**

The roflumilast treatment outcomes comparing BD and RAS group. The error bars indicate the standard error. (a) Response rate evaluated at weeks 4,8 and 12. (b) Number of current lesions at the visit. (c) Total number of oral ulcers during 4-week period. (d) Average duration of an individual oral ulcer lesion. (e) Total duration of overall oral ulcers within 4-week period. (f) Pain score evaluated by NRS score at each evaluation. Statistical comparison was evaluated comparing BD and RAS group for each week 4, 8 and 12. (Abbreviations: BD, Behçet’s disease; RAS, recurrent aphthous stomatitis; NRS, numeric rating scale; NS, no statistical difference)

**
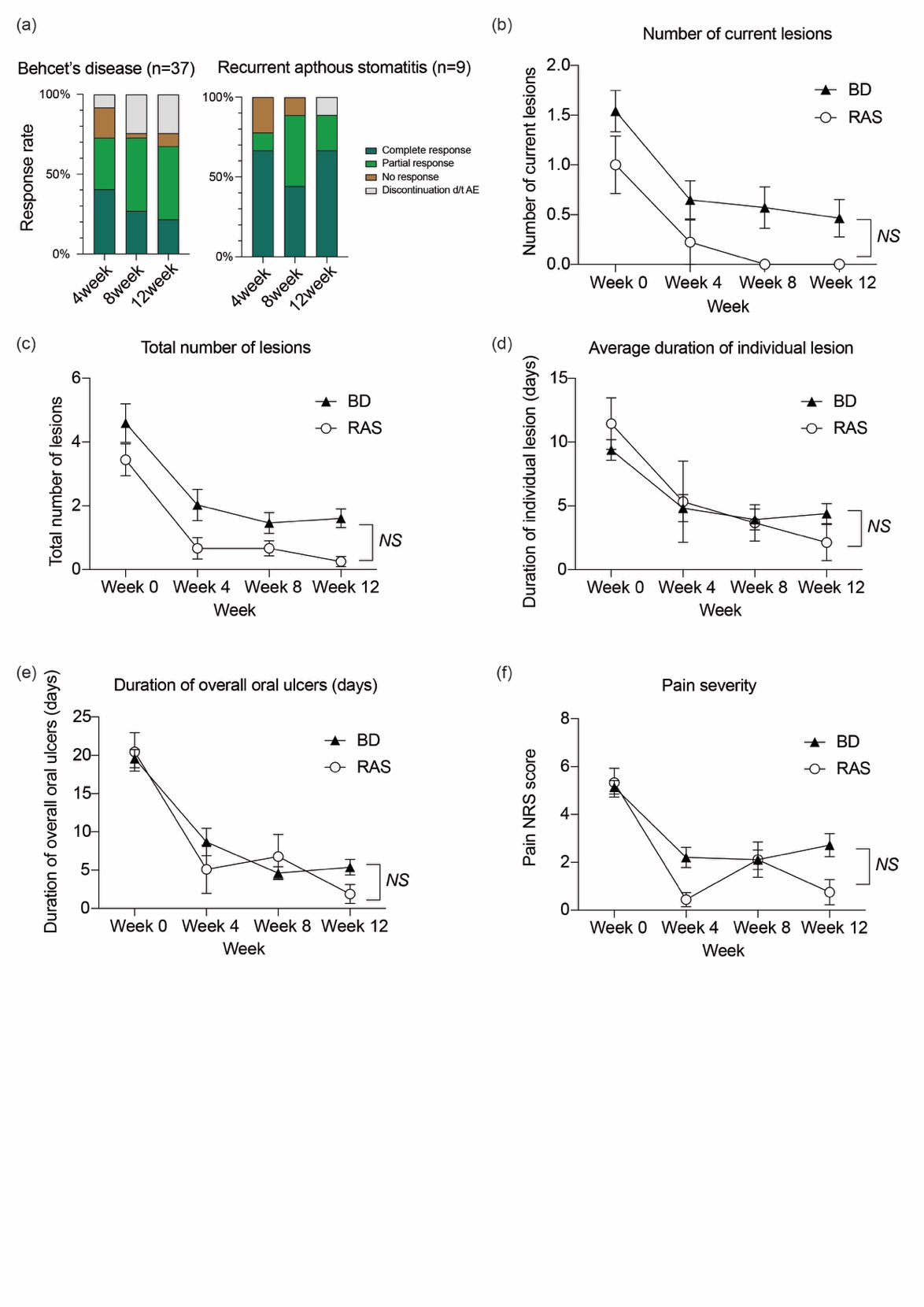
**

**Supplemental Table 1. Treatment outcomes after administration of roflumilast.**

| Total subjects (N=46) | Week 0 | Week 4 | Week 8 | Week 12 |
| --- | --- | --- | --- | --- |
| Primary outcome, No. (%) |  |  |  |  |
| Complete remission (CR) | *NA* | 21 (45.7%) | 14 (30.4%) | 14 (30.4%) |
| Partial response (PR) | *NA* | 13 (28.3%) | 21 (45.7%) | 19 (41.3%) |
| Non-responder (NR) | *NA* | 9 (19.6%) | 2 (4.4%) | 3 (6.5%) |
| Discontinuation due to intolerability | *NA* | 3 (6.5%) | 9 (19.6%) | 10 (21.7%) |
| Secondary outcome (Mean, SD) |  |  |  |  |
| No. of current lesions | 1.44 (1.21) | 0.56 (1.05) | 0.43 (0.98) | 0.36 (0.90) |
| Total No. of lesions (in 4 weeks) | 4.37 (3.40) | 1.74 (2.63) | 1.27 (1.56) | 1.31 (1.49) |
| Average duration of the single lesion (days) | 9.78 (5.10) | 4.93 (6.90) | 3.87 (4.23) | 3.89 (4.21) |
| Lesion presence period (in 4 weeks, days) | 19.74 (7.26) | 8.58 (10.63) | 5.14 (5.72) | 4.61 (5.26) |
| Pain (NRS) | 5.17 (1.70) | 1.84 (2.34) | 2.11 (2.15) | 2.28 (2.49) |

Abbreviations**:** NRS, Numeric rating scale;
